# Photoelectrochemical photocurrent switching effect on a pristine anodized Ti/TiO_2_ system as a platform for chemical logic devices†

**DOI:** 10.1039/d0ra00205d

**Published:** 2020-03-26

**Authors:** Nikolay V. Ryzhkov, Veronika Yu. Yurova, Sviatlana A. Ulasevich, Ekaterina V. Skorb

**Affiliations:** ITMO University 9, Lomonosova street Saint Petersburg 191002 Russia skorb@itmo.ru

## Abstract

We report here the effect of the photoelectrochemical photocurrent switching (PEPS) observed on highly-ordered pristine anodized Ti/TiO_2_ for the first time. At negative potential bias, blue irradiation gives cathodic photocurrent, whereas anodic photocurrent was observed for ultraviolet irradiation. We believe this phenomenon is due to the electron pathway provided by Ti^3+^ defect states.

## Abstract 2

We report here the effect of the photoelectrochemical photocurrent switching (PEPS) observed on highly-ordered pristine anodized Ti/TiO_2_ for the first time.

## Graphical abstract

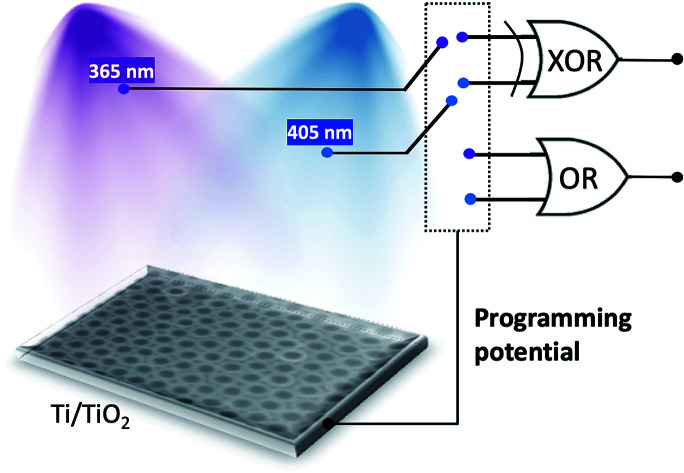

Titanium dioxide, being one of the most studied materials, still draws much attention from researchers.^1,2^ It is considered to be a very promising material due to its high chemical stability, nontoxicity, and its unique properties. Due to stable and robust photoactivity, titania is widely used in the design of solar cells^3^ and photocatalytic applications.^4^ In addition to the fact that titanium dioxide occurs in several crystalline modifications, it can also be obtained in various forms, such as, for example, nanotubes,^5^ nanofibers,^6^ and nanosheets.^7^ The photocatalytic performance of TiO_2_ is highly dependent on crystallinity,^8^ phase content, form, and preparation method.^9^ It was reported that highly ordered arrays of TiO_2_ nanotubes are characterized by short charge transport distance and little carrier transport loss.^5^ Therefore, electrochemically fabricated TiO_2_ nanotube arrays are preferable compared to random non-oriented titania.^10^ Great varieties of photoelectrochemical behaviour can be achieved by doping^11^ and surface modification.^12,13^

An interesting feature has recently been demonstrated for highly ordered arrays of TiO_2_ nanotubes obtained by double stepwise electrochemical anodization of a titanium foil (Ti/TiO_2_). Together with our colleagues observed that localized illumination of Ti/TiO_2_ surface in water solution triggers proton flux from irradiated area.^14^ The photocatalytic activity of TiO_2_ is based on photogenerated electron–hole pairs. Under the electric field of Ti/TiO_2_ Schottky junction and due to upward surface band bending, efficient spatial charge separation occurs, and photoexcited holes (h^+^) reach TiO_2_ – solution interface. The h^+^, which is a strong oxidizing agent, can react with water, and a pronounced pH gradient arises due to water photolysis. Thus, titanium dioxide can be used to trigger local ion fluxes, and proton release is associated with anodic photocurrent. The use of the light-pH coupling effect to control pH-sensitive soft matter was previously demonstrated.^15,16^ Complementary species, H^+^ and OH^−^, annihilating when occurring simultaneously, extend chemical arithmetic with subtraction operation opening way to pure chemical calculations.^17^ Ion fluxes consideration as information transducers in solution were proposed^18^ and performing simple logic operations was demonstrated.^19^ This phenomenon opens perspectives to biomimetic information processing and developing effective human–machine interfaces.^20^

Photoelectrodes using light and potential as inputs and yielding photocurrents are being considered as the basis for logic devices. In this way, optical computing compatible with existing silicon-based devices may be performed.

Logic operations are described by Boolean algebra operating with truth values denoted 0 (false) and 1 (true). Elementary logical operations are modelled by logic gates producing single binary output from multiple binary inputs and physically implemented by some switch. As for photoelectrode based information processing, the photoelectrochemical photocurrent switching (PEPS) effect is utilized. This effect is that under appropriate external polarization or/and illumination by light with appropriate photon energy, switching between anodic and cathodic photocurrent may be observed for n-type semiconductors and the opposite for p-type.^21,22^

Without further modification, this effect was observed for a very limited number of materials, such as bismuth orthovanadate, lead molybdate, V–VI–VII semiconductors, and some others. To show this effect, the majority of semiconductors require electronic structure perturbation creating new electron pathways. A convenient solution is specific modifier adsorption onto the semiconductors' surface, providing a sufficient level of electronic coupling. Photoelectrodes made of nanocrystalline TiO_2_ modified by cyanoferrate,^13,23^ and ruthenium^24^ complexes, thiamine, folic acid,^25^ and carminic acid^26^ demonstrated PEPS behavior.

Surprisingly, we observed the PEPS effect on non-modified Ti/TiO_2_ obtained by anodation of Ti plates.

Highly ordered arrays of anatase Ti/TiO_2_ were obtained. Crystallinity was proved by XRD (Fig. S1a†). Fig. 1a shows a SEM image of TiO_2_ nanotube arrays obtained as described above. According to SEM image, an average pore diameter is *ca.* 60 nm. As reported, highly ordered TiO_2_ nanotubes possess a short charge transport distance and little carrier transport loss. Therefore, highly ordered TiO_2_ nanotube arrays fabricated by electrochemical anodization of titanium may exhibit some enhanced capacity of electron transfer than non-oriented ones of random mixture.^10^

**Fig. 1:**
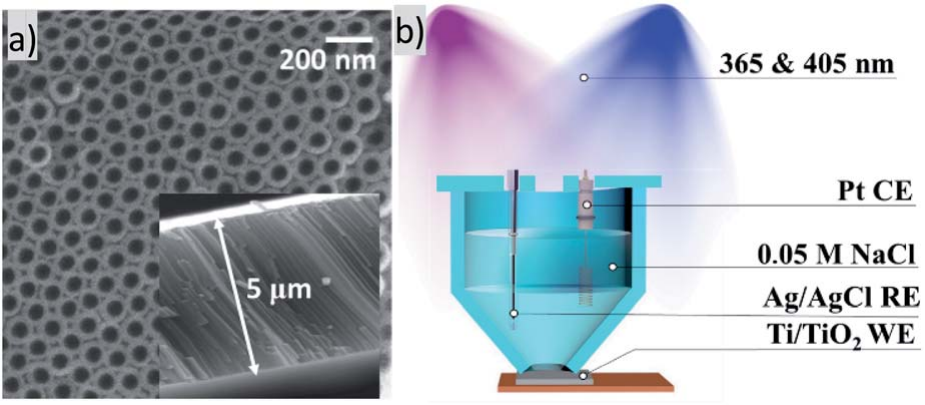
(a) SEM image of the TiO_2_ nanotubes array. The inset shows cross-section view. (b) Scheme of a cell for photocurrent measurements experiment, CE – counter electrode, RE – reference electrode, WE – working electrode.

According to Mott–Schottky analysis, at potential bias more positive than −0,697 V *vs.* Ag/AgCl reference electrode upwards band bending occurs (Fig. S2†). Heat treatment in a nonoxidizing atmosphere leads to Ti^3+^ formation. Appearance of Ti^3+^ self-doping was proved by EDX analysis (Fig. S1b†). It was previously reported that Ti^3+^ introduces gap states which act as recombination centers and pathways for electron transfer.^27–29^ Ti^3+^ species in reduced TiO_2_ introduce a gap state between valence and conduction bands.^27,28^

We studied dependence of photocurrent on applied potential. Ultraviolet irradiation (365 nm) gave positive photocurrent for all potentials studied in range from −0.6 V to 0.6 V *vs.* Ag/AgCl reference electrode (Fig. S3†). The photocurrent increases as the potential becomes more positive, but eventually saturates. The dependence of the current on the potential under blue irradiation (405 nm) had a different character. Sigmoid function with inflection point at 0–0.2 V was observed for blue light.

It should be noticed that photocurrent plotted against time on Fig. 2–4 as well as against potential on Fig. S3† is Δ*I* = *I*_under illumination_ − *I*_in darkness_. Steady state current values were used for calculations.

**Fig. 2:**
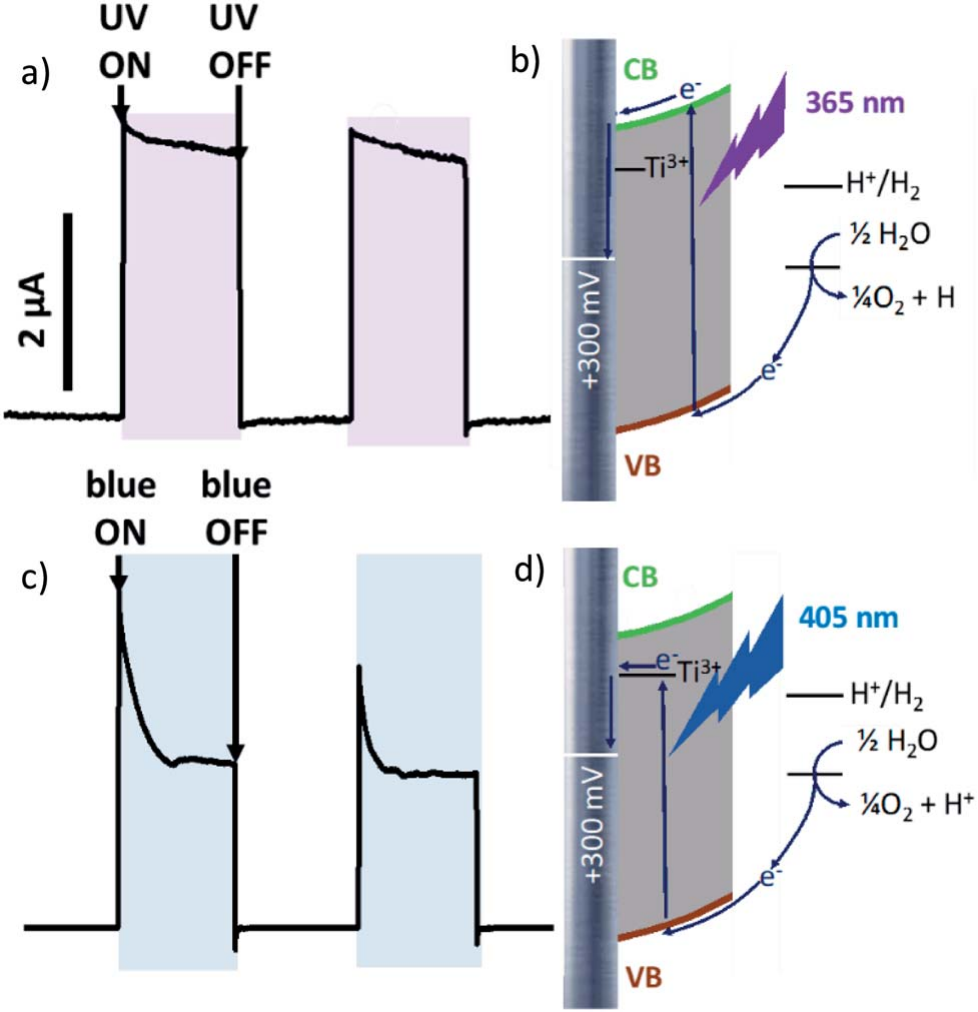
Photocurrent curves under chopped irradiation by (a) 365 nm UV LED, (c) 405 nm blue LED at applied potential bias +300 mV *vs.* Ag/AgCl, and corresponding scheme of electron pathway at +300 mV polarization under irradiation by (b) 365 nm UV LED and (d) 405 nm blue LED.

**Fig. 3:**
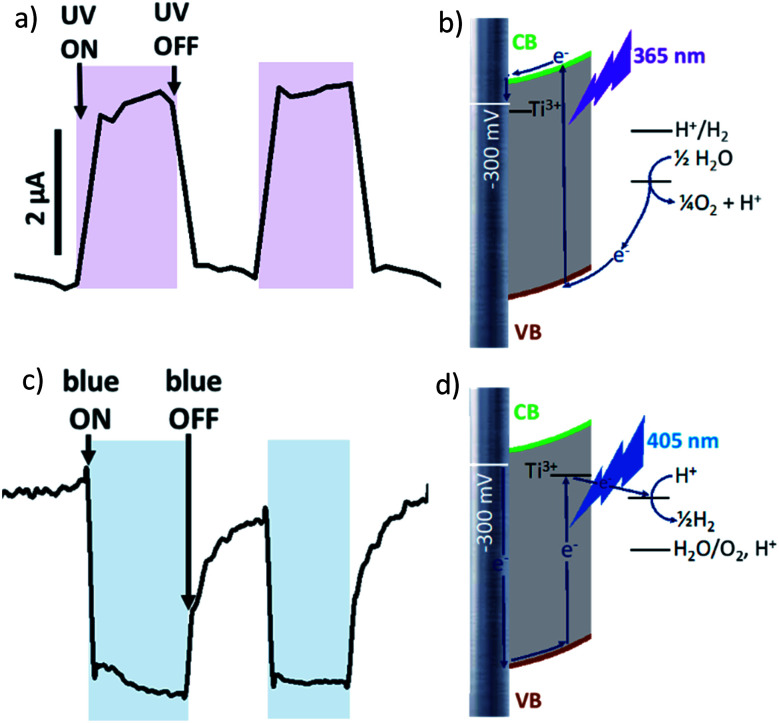
Photocurrent curves under chopped irradiation by (a) 365 nm UV LED, (c) 405 nm blue LED at applied potential bias −300 mV *vs.* Ag/AgCl, and corresponding scheme of electron pathway at −300 mV polarization under irradiation by (b) 365 nm UV LED and (d) 405 nm blue LED.

**Fig. 4:**
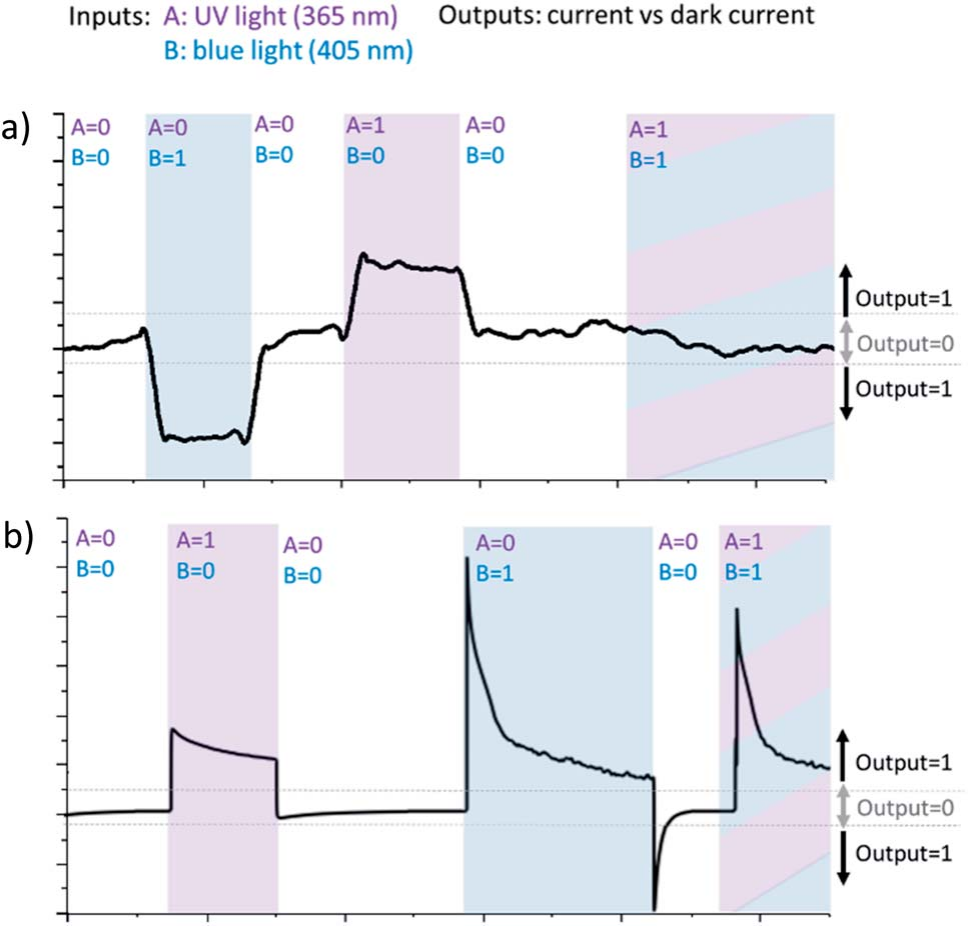
(a) XOR logic realized on negatively polarized (−0.3 V) pristine Ti/TiO_2_ by two source irradiation, input A – UV light (365 nm), input B – blue light (405 nm); blue light gives anodic photocurrent, UV – cathodic photocurrent. The current, significantly different from the dark one, is taken as output 1, otherwise – 0. When irradiated by blue and UV light simultaneously, anodic and cathodic current compensate each other, and no total photocurrent observed. Thus output 0, when both inputs are 1 (b) OR logic realized on positively polarized (+0.3 V) non-modified Ti/TiO_2_ by two sources of irradiation. Irradiation by any of them, blue or UV, gives anodic photocurrent.

At +300 mV *vs.* Ag/AgCl irradiation by both blue and ultraviolet light give anodic photocurrent (Fig. 2a and c). The UV-irradiation (*λ* = 365 nm, 5 mW cm^−2^) excites electron directly to the conduction band (CB) of TiO_2,_ which is further transferred to conducting titanium support (Fig. 2b). When Ti/TiO_2_ electrode in thermodynamic equilibrium with electrolyte, an upward surface band bending occurs at the semiconductor–liquid junction. This phenomenon obstructs electron injection from the conduction band into the electrolyte and forces electron drift to conducting substrate. The fast and steady photocurrent production/extinction upon light on/off indicates efficient charge separation and low recombination.

Blue light (*λ* = 405 nm, 70 mW cm^−2^) is characterized by lower energy than UV-irradiation, which is not sufficient to excite the electron to CB. But electron excited by blue light can be trapped by Ti^3+^ located close to the conduction band and transferred to conduction support from these levels (Fig. 2c). An initial current spike following by an exponential decrease suggesting a fast recombination process. It should be also noticed than when irradiation is switched off photocurrent ‘overshoots’ as the remaining surface holes continue to recombine with electrons.

At more negative potential (−300 mV *vs.* Ag/AgCl, for example) applied to non-modified anodized Ti/TiO_2_ photoelectrode, we observed anodic photocurrent during irradiation by UV light (Fig. 3a) whereas blue irradiation gave anodic photocurrent (Fig. 3c). Excitation within bandgap by UV-irradiation leads to cathodic photocurrent (Fig. 3b). In the case of irradiation by blue light, electron trapping by Ti^3+^ occurs in the same manner as at +300 mV polarization. But at negative polarization, the energy landscape is such that electron transport to electron donor in solution is preferable (Fig. 3d). As a result, cathodic current occurs.

Thereby, photoelectrode activity of non-modified anodized Ti/TiO_2_ can be switched from anodic to cathodic and *vice versa* by applying various potentials and various photon energies. This is the effect of photoelectrochemical photocurrent switching.

Thereby, when Ti/TiO_2_ is irradiated simultaneously by blue and UV light being negatively polarized, competition between cathodic and anodic photocurrents occurs. Returning to Boolean logic, the PEPS effect allows us to perform annihilation of two input signals and implement optoelectronic XOR logic gate. XOR logic operation outputs true (1) only when input values are different and yield zero otherwise.

It is necessary to assign logic values to input and output signals to analyse the system based on Ti/TiO_2_ PEPS effect in terms of Boolean logic. Logical 0 and 1 are assigned to off and on states of the LEDs, respectively. Different wavelengths (365 and 405 nm) correspond to two different inputs of the logic gate. In the same way, we can assign logic 0 to the state when photocurrent is not generated and logic 1 to any nonzero photocurrent intensity irrespectively on its polarization (cathodic or anodic).

Fig. 4 demonstrates how different types of Boolean logic are realized by irradiation of Ti/TiO_2_. Light sources are denoted here as inputs, UV light – A and blue light – B. If the corresponding light source is switched ON and illuminates photoelectrode Ti/TiO_2_, this input is ‘1’, otherwise, it's ‘0’. The photocurrent is read as output. It's considered to be ‘1’ if significantly differs from dark value and ‘0’ otherwise.

At −300 mV *vs.* Ag/AgCl, pulsed irradiation with UV diode (365 nm, 5 mW cm^−2^) results in anodic photocurrent, which is consistent with electron excitation to CB and transfer to conducting support. Irradiation with blue LED (405 nm) gives cathodic photocurrent due to electron capture by Ti^3+^ states following by transferring to electron acceptor in solution. Simultaneous irradiation with two LEDs with adjusted intensity yields zero net current as anodic and cathodic photocurrents compensate effectively (Fig. 4a).

At positive potentials, pulsed irradiation with UV diode gives anodic photocurrent pulses, as well as the blue one. It is interesting to note that when two sources of light are simultaneously irradiated, the photocurrents created by each of them individually do not summarize. At +300 mV, photocurrent output under the influence of two light inputs (365 nm and 405 nm) follows OR logic giving positive output if at least one of inputs is positive (Fig. 4b).

Fig. 5 demonstrates the reconfigurable logic system which characteristics can be changed *via* an appropriate polarization of the photoelectrode regarded as programming input. Two irradiation sources are considered as inputs. OR/XOR logic is realized depending on programming input.

**Fig. 5:**
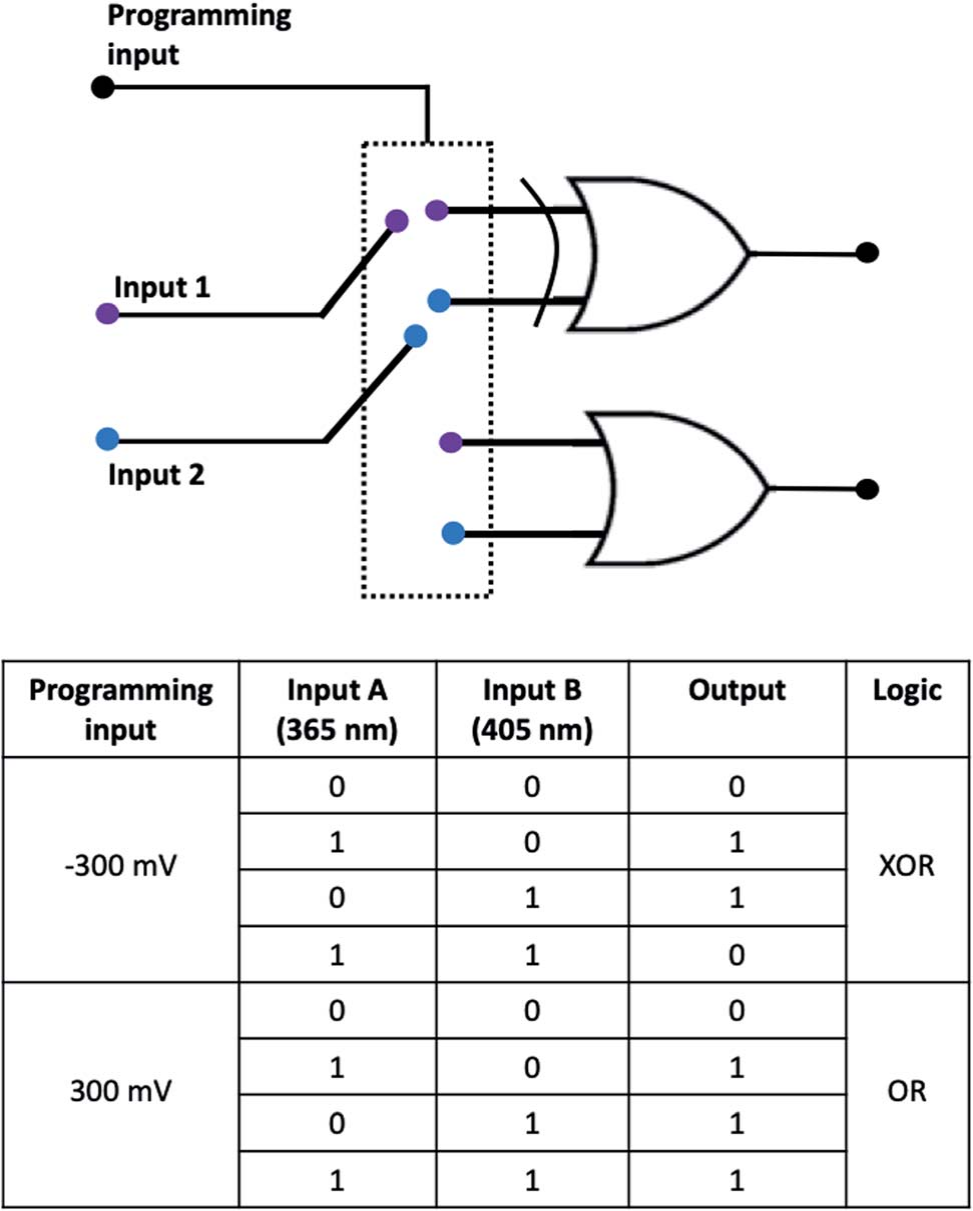
A reconfigurable logic system based on non-modified Ti/TiO_2_. Light sources are inputs. The choice between XOR and OR function is determined by programming input of potential bias. At +300 mV OR logic is realized, at −300 – XOR logic. Corresponding truth table is presented.

In summary, PEPS effect on modified nanocrystalline TiO_2_ was previously discussed a lot.^13,23–26^ In this work we report the same phenomenon for pristine anodized Ti/TiO_2_ system. Due to substructure of Ti/TiO_2_ system, it shows characteristic response to various range of illumination, including visible range and polarization. The Ti/TiO_2_ system is a simple and robust model of chemical logic gates. Suggested mimicking of logic functions in aqueous solutions allows further integration of element into communication with living objects^16^*vs.* intrinsically associated photooxidation and degradation, but rather activation for needed function.^30^

## Experiment

Highly ordered arrays of photoactive crystalline TiO_2_ nanotubes can be obtained by double two-stage anodization of titanium substrates in ethylene glycol electrolyte containing fluoride ions. Double anodization involves forming a first anodization layer and an adjacent second anodization layer on an angled surface, the interface between the two anodization layers being regular and uniform. Anodization was performed *via* two stages where at the first stage the anode was linearly polarized from 0 to 40 V, and then at the second stage, the electrode was polarized at a constant voltage of 40 V. The as prepared Ti/TiO_2_ were treated ultrasonically in ethanol for 0.5–1 min to remove the debris and annealed at 450 °C for 3 hours.

For Mott–Schottky analysis potential was scanned from −1.0 to +0.4 with increment 0.04 V and 3 minutes delay for equilibration of each potential. Potential were oscillating with amplitude 0.005 V and frequency 1000 Hz.

Photocurrent measurements were performed in 3-electrode cell with Ti/TiO_2_ nanotubes plate as working electrode, Pt counter electrode and Ag/AgCl reference electrode. During each measurement desired potential bias was applied and after establishing a constant stable dark current working electrode was illuminated by UV LED (365 nm) or blue LED (405 nm) or both light sources simultaneously.

For Boolean logic implementation light intensity was adjusted the light intensity was adjusted in such a way that under −0.3 V polarization photoresponses on UV and blue light annihilate.

## Conflicts of interest

There are no conflicts to declare.

## Supplementary Material

RA-010-D0RA00205D-s001
