# Supplementary material for: Photoelectrochemical photocurrent switching effect on a pristine anodized Ti/TiO2 system as a platform for chemical logic devices
Source: RSC Adv. 2020 Mar 26;10(21):12355–9. doi: 10.1039/d0ra00205d (PMC9050699; doi:10.1039/d0ra00205d)
Supplement: RA-010-D0RA00205D-s001 [file RA-010-D0RA00205D-s001.pdf]

Electronic Supplementary Material (ESI) for RSC Advances.
This journal is © The Royal Society of Chemistry 2020

Supporting Information

Photoelectrochemical Photocurrent Switching Effect on Pristine Anodized Ti/TiO_2 System as a Platform for Chemical Logic Device

Nikolay V. Ryzhkov, Veronika Yu. Yurova, Sviatlana A. Ulasevich and Ekaterina V. Skorb *

ITMO University, Lomonosova str. 9, 191002 Saint Petersburg, Russia
*E-mail: skorb@itmo.ru

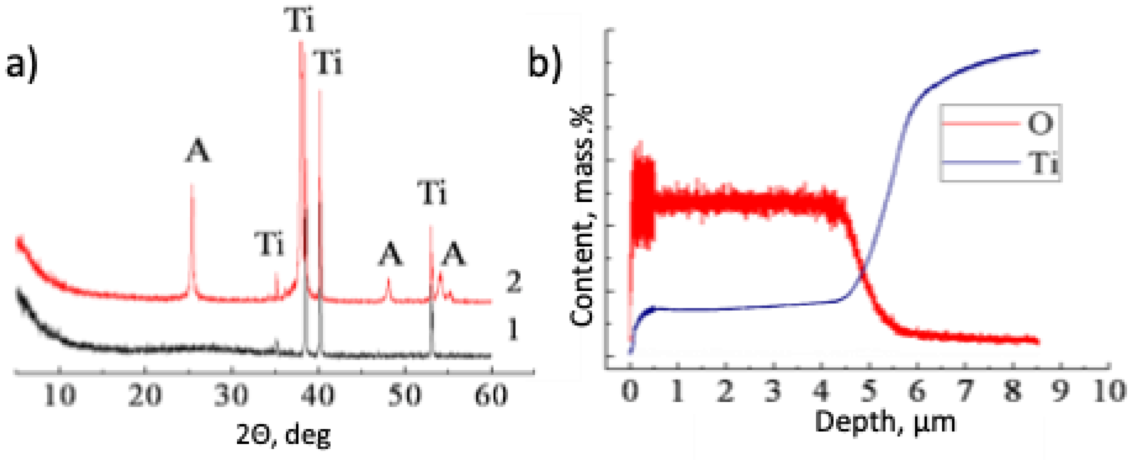

Figure S1. a) XRD patterns of anodized Ti/TiO_2 nanotubes without annealing (curve 1) and after their heat treatment at 450°C (curve 2), peaks appear in the 2Θ region: 25.18; 37.81; 48.01; 54.01 and 55.08, which correspond to the peaks of the anatase modification of TiO_2, b) EDX demonstrating Ti/O ratio in the resulting Ti/TiO_2 nanotubes depending on depth (as you approach the titanium substrate). Ti:O varies on average from 1: 1.7 to 1: 1.9, which is close to the Ti/O ratio in the TiO_2 phase and evidences Ti^{3+} self-doping.

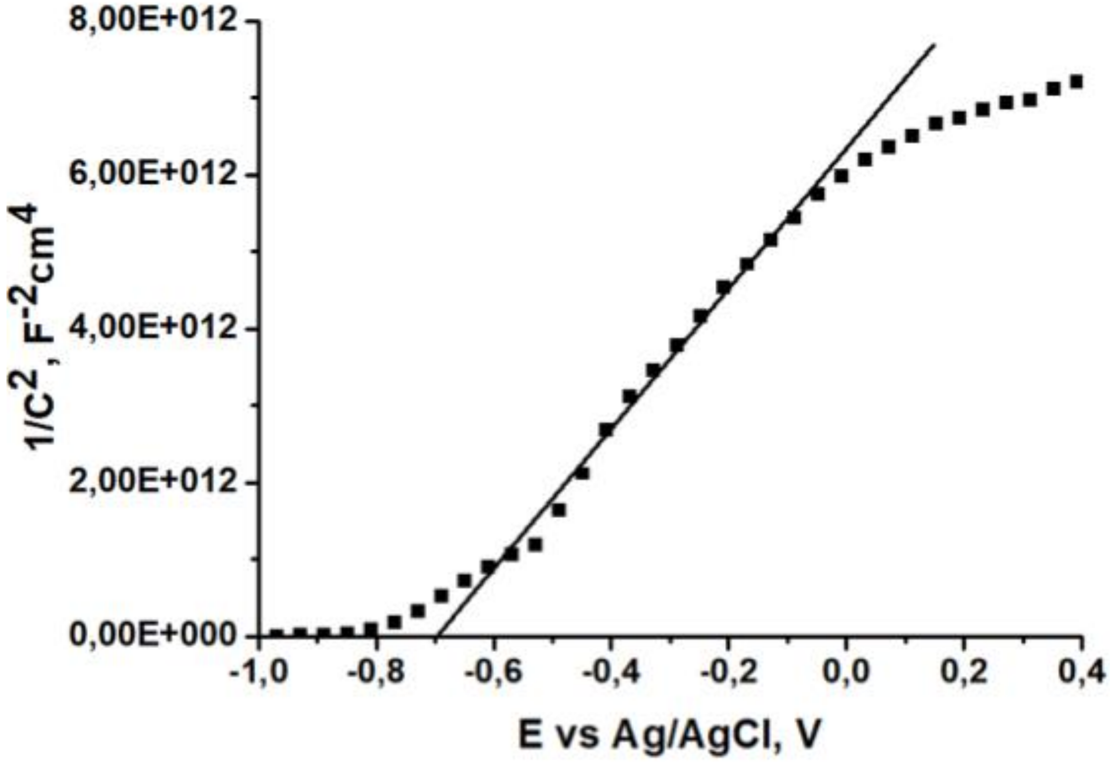

Figure S2. Mott-Schottky plot for anodized pristine Ti/TiO_2 nanotubes.

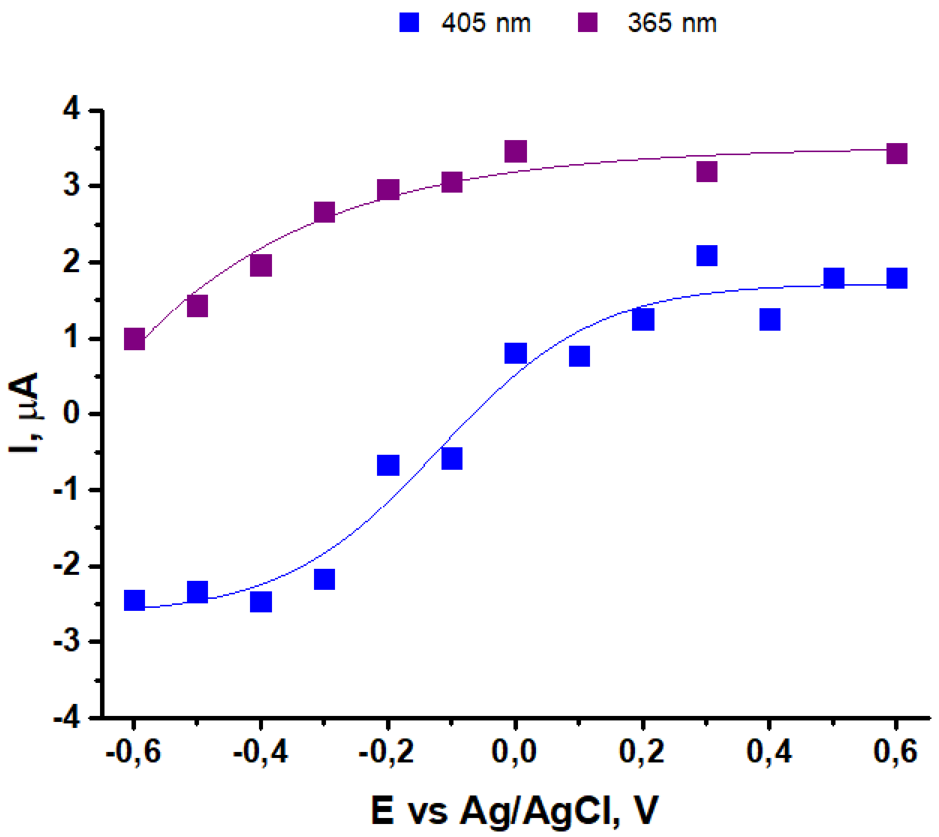

Figure S3. Photocurrent dependence on applied potential for ultraviolet irradiation (365 nm) – violet line and blue irradiation (405 nm) – blue line.
